# Excess Conductivity Analysis of Polycrystalline FeSe Samples with the Addition of Ag

**DOI:** 10.3390/ma13215018

**Published:** 2020-11-06

**Authors:** Michael Rudolf Koblischka, Yassine Slimani, Anjela Koblischka-Veneva, Thomas Karwoth, XianLin Zeng, Essia Hannachi, Masato Murakami

**Affiliations:** 1 Experimental Physics, Saarland University, P.O. Box 151150, D-66044 Saarbrücken, Germany; anjela@shibaura-it.ac.jp (A.K.-V.); thomaskarwoth@icloud.com (T.K.); x.zeng@physik.uni-saarland.de (X.Z.); 2 SIT Research Laboratories, Shibaura Institute of Technology, Tokyo 135-8548, Japan; masatomu@shibaura-it.ac.jp; 3 Department of Biophysics, Institute for Research and Medical Consultations (IRMC), Imam Abdulrahman Bin Faisal University, P.O. Box 1982, Dammam 31441, Saudi Arabia; yaslimani@iau.edu.sa; 4 Laboratory of Physics of Materials—Structures and Properties, Department of Physics, Faculty of Sciences of Bizerte, University of Carthage, Zarzouna 7021, Tunisia; hannechi.essia@gmail.com

**Keywords:** iron-based superconductors, FeSe, silver addition, microstructure, resistance, fluctuation-induced conductivity

## Abstract

Bulk FeSe superconductors of the iron-based (IBS) “11” family containing various additions of silver were thoroughly investigated concerning the microstructure using optical microscopy and electron microscopy (TEM and SEM). The measurements of electrical resistivity were performed through the four-point technique in the temperature interval T= 2–150 K. The Aslamazov–Larkin model was employed to analyze the fluctuation-induced conductivity (FIC) in all acquired measurements. In all studied products, we found that the FIC curves consist of five different regimes of fluctuation, viz. critical region (CR), three-dimensional (3D), two-dimensional (2D), one-dimensional (1D), and shortwave fluctuation (SWF) regimes. The critical current density ($J_{c}$), the lower and upper critical magnetic fields ($B_{c1}$ and $B_{c2}$), the coherence length along the *c*-axis at zero-temperature ($\xi_{c}$(0)), and further parameters were assessed with regards to the silver amount within the products. The analyses discloses a diminution in the resistivity and a great reduction in $\xi_{c}$(0) with Ag addition. The optimal silver doping amount is achieved for 7 wt.%, which yields the best superconducting transition and the greatest $J_{c}$ value.

## 1. Introduction

Polycrystalline, iron-based superconductors (IBS) are interesting candidates for several applications of superconductivity at low temperatures [1,2,3]. This is owed to the relative simplicity, the good grain coupling, and their behavior and parameters, analogous to the cuprate high-temperature superconductor (HTSc) systems, which overcome the flux-jump problem observed in the MgB${}_{2}$ bulks [4]. Thus, the IBS materials claim their place in the form of superconducting wires or tapes [2], as well as bulk materials intended for trapped-field applications [5].

The simplest compound of the iron-based superconductors, FeSe (abbreviated “11”) [6,7], is also an interesting material for applications [1,8]. As a polycrystalline material, the 11 material offers a simple production technology and does not contain expensive rare earth materials or toxic elements, and the grain boundaries (GBs) are assumed not to act as weak-links like in cuprate HTScs [9,10,11]. This makes the 11 compounds interesting for bulk applications like superconducting trapped-field magnets or “super magnets” [12]. Furthermore, even though the iron-based superconductors are materials with relatively low superconducting transition temperatures, $T_{c}$, the superconducting properties like the high upper critical fields, $B_{c2}$, are comparable to other HTSc systems, being much better, as for the direct metallic competitor, MgB${}_{2}$. These advantages were already demonstrated for the IBS 122 compounds in the literature [5,13,14,15], but not yet for the 11 system.

However, the preparation of bulk samples of the 11 family is not straightforward due to the quite complicated phase diagram [16]. There are several competing phases, α-FeSe, δ-FeSe, the superconducting β-FeSe, antiferromagnetic Fe${}_{7}$Se${}_{8}$, and also α-Fe. The preparation route firstly creates δ-FeSe at temperatures above 750 ${}^{\circ }$C, which is then converted to β-FeSe during the cooling process. The stoichiometry is quite critical [17,18,19], as excess Fe leads to a ferromagnetic sample, and excess Se may favor the formation of Fe${}_{7}$Se${}_{8}$. Furthermore, the resulting FeSe samples are quite porous [20], so it is interesting to prepare polycrystalline, bulk FeSe samples with the addition of metallic silver, which can effectively fill the pores and reduce the sample resistivity. Such experiments were already performed in the literature by several groups [21,22], and it also became clear that Ag may enter the FeSe unit cell and, thus, influence the superconducting properties. Thus, a proper analysis of the resulting superconducting traits of Ag-FeSe systems is needed.

For the excess conductivity or fluctuation-induced conductivity (FIC), various model approaches have been proposed such as Aslamazov–Larkin (AL) [23], Lawrence–Doniach (LD) [24], and Maki–Thompson (MT) models [25]. FIC was already broadly investigated for many HTSc materials in the literature, and the majority of them are based on AL model analysis. The short coherence length, high anisotropy, and lower charge carrier density of HTSc materials cause an apparent rounding of the measured curves of resistivity near the transition to the superconducting state [26]. This enables the investigation of the fluctuations of superconducting Cooper pairs in a wide range of temperature above $T_{c}$. These analyses are the key to obtain knowledge concerning the microscopic characteristics of the HTSc systems like the order parameter’s dimensionality, the *c*-axis coherence length, the crossover temperatures, the critical current density $J_{c}$, the critical fields $B_{c1}$, $B_{c2}$, etc. [26,27]. Moreover, theoretical predictions of the critical region (CR) close to $T_{c}$ or of the construction of Cooper pairs can be studied via resistivity measurements. The FeSe superconductors do not have Cu-O planes, but a layered 2D structure, so the LD approach is also valid here.

In the present contribution, we thoroughly investigate the microstructure of polycrystalline and bulk FeSe samples with the addition of silver and employ the analysis of the excess conductivity or fluctuation-induced conductivity (FIC) to the FeSe-Ag system.

## 2. Experimental Procedures

### 2.1. Sample Preparation and Characterization

The bulk, polycrystalline FeSe material was produced at Shibaura Institute of Technology (SIT), Tokyo, using a solid-state sintering route [20,22,28]. Commercially available high purity powders of Se (99.5%) and Fe (99.9%) were employed. Inside a glove box, these powders were initially mixed according to the stoichiometric ratio Se:Fe = 1:1 and completely ground in an agate mortar, inserted inside a ceramic jar under an argon atmosphere (with high purity), and then, ball milled for 6 h. The resulting powder was pressed into discs (height = 2 mm and diameter = 5 mm). The samples were packed inside an evacuated quartz tube and sealed. Then, the arrangement was heat-treated for 24 h at 600 ${}^{\circ }$C. Afterwards, the obtained discs were again completely ground and followed by a second ball milling stage. The final products were again sealed in a quartz tube and heat-treated at two distinct temperatures for 24 h, and at the end, the temperature was cooled to ambient temperature at a rate of 230 ${}^{\circ }$C/h. The pristine polycrystalline bulk FeSe product was produced at reaction temperatures of 900 and 950 ${}^{\circ }$C, whereas products added with different amounts of silver were heated at a lower temperature of 825 ${}^{\circ }$C. More details about the preparation protocol can be found in [20,22].

The component phases of the product were recognized via an Rigaku (Tokyo, Japan) RINT 2200 X-ray powder diffractometer (Cu-Kα radiation, 50 kV, 300 mA). Polarization photos were done by a Zeiss (Oberkochen, Germany) polarization microscope. Scanning electron microscopy (SEM) images were taken by a Hitachi (Hitachi High-Tech Ltd., Tokyo, Japan) S800 SEM working at a voltage of 20 kV. Atomic force microscopy (AFM) topography images were recorded in the tapping mode of a Veeco-DI Nanoscope IV (Plainview, NY, USA) with a Q-control unit.

### 2.2. Resistance Measurements

The resistance experiments were recorded in the temperature interval of 2–300 K (with a step of 0.25 K) using a 10–12 T Teslatron cryostat from Oxford Instruments (Oxford, UK) equipped with a Keithley 2400 source-meter (Solon, OH, USA) and a temperature controller “ITC 4” (Oxford Instruments, Oxford, UK). For that, rectangular bars were cut from the main discs of 1 × 2 × 5 mm${}^{3}$ in dimensions. The measurements were performed through the four-probe technique. Accordingly, for lower resistance contacts, very thin Cu wires were utilized connected on the surface of the sample with Ag paste [28]. The magnetic field was applied perpendicular to the long surface of the sample. The registration of resistivity was conducted using a customized MATLAB (MathWorks, Natick, MA, USA) program [28,29].

## 3. Excess Conductivity

The excess conductivity (EC) caused by thermal fluctuations of the order parameter, ψ, of HTSc systems is influenced by both the internal and extrinsic characteristics of the product, including structural defects, the morphologies of grains, the grain coupling, etc. The EC, denoted Δσ, is determined via:

(1) $$\Delta \sigma =\frac{1}{\rho (T)}-\frac{1}{\rho_{n}\left(T\right)}\;\;\;,$$

where ρ(T) is the experimental resistivity and $\rho_{n}\left(T\right)$ is the resistivity in the normal state. According to the AL theory [23], Δσ in the mean-field regime (MFR) above $T_{c}$ (here, the mean-field temperature, $T_{c}$, corresponds to the temperature at which the dρ/d*T* curve shows a peak) is specified by: $\Delta \sigma =A\epsilon^{-\lambda }$, wherein $\epsilon =\left(T-T_{c}\right)/T_{c}$ is the reduced temperature and the exponent λ is correlated to the dimensionality of conduction D. Thus, the conductivity exponent λ can take the values 0.33 for dynamic fluctuations ($\lambda_{\mathrm{CR}}$), 0.66 for static critical fluctuations, 0.5 for 3D fluctuations, 1.0 for 2D fluctuations, 1.5 for 1D fluctuations, and finally, 3.0 for shortwave fluctuation (SWF). The amplitude *A* is expressed by:

(2) $$A=\frac{e^{2}}{32\hbar \xi_{c}\left(0\right)}\;\left(3D\right),\;\;A=\frac{e^{2}}{16\hbar d}\;\left(2D\right),\;\;A=\frac{e^{2}\xi_{c}\left(0\right)}{32\hbar s}\;\left(1D\right)\;\;,$$

for respectively the 3D, 2D, and 1D fluctuations. *s* is the wire cross-sectional area of 1D systems. *d* is the thickness of the effective layers of 2D systems. $\xi_{c}\left(0\right)$ is the *c*-axis coherence length at 0 K. *e* is the electron charge.

By plotting ln(Δσ) against ln(ϵ), various fluctuation regimes by linear fitting along with the exponent values (from the slopes) will be determined.

## 4. Results and Discussion

### 4.1. Microstructure

**Figure 1:**
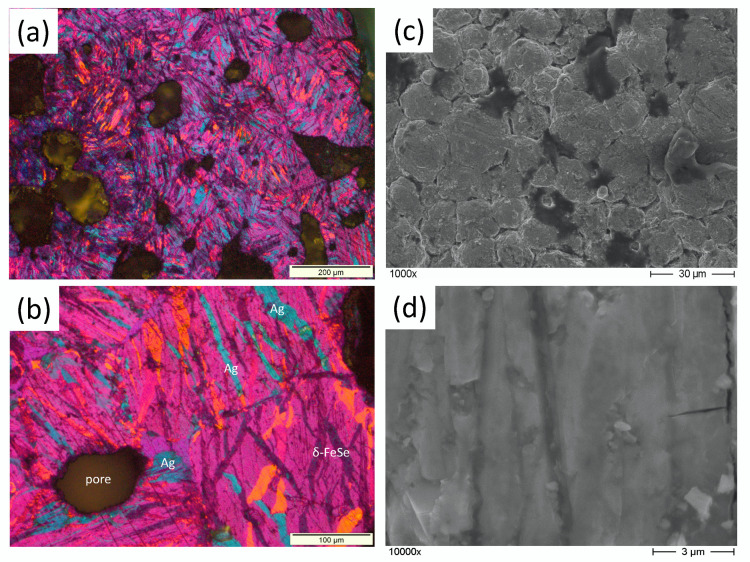
(**a**,**b**) Polarization images of the 4%-FeSe+Ag-sample. (**c**,**d**) SEM images of the same sample. The polarization images directly reveal the various phases and give an impression of the porosity. The metallic Ag particles appear in light blue color; the remaining δ-FeSe phase is imaged in dark grey; and the different orientations of the superconducting β-phase are in reddish colors. Note also the typical acicular shape of the δ-FeSe grains. The α-Fe and Fe${}_{7}$Se${}_{8}$ particles are too small to be seen in the optical images.

The microstructural investigations of the products were investigated by polarization microscopy and SEM imaging. Figure 1a,b shows typical polarization images of the product added with Ag content of 4 wt.%. Here, Ag particles appear light blue; the remaining δ-FeSe phase is imaged in dark grey; and the different orientations of the superconducting β-FeSe phase are in reddish colors. The Ag largely tends to occupy the grain boundaries and fills some of the pores in the FeSe grain structure. Furthermore, several pores are still visible in the images. Particles of α-Fe and Fe${}_{7}$Se${}_{8}$ are too small to be seen in the optical images, but could be detected in electron backscatter diffraction analysis [30]. Therefore, it is obvious that the superconducting transport currents have to cross the various grain boundaries and to circumvent the non-superconducting phases and the pores present in the sample. This will be important for the interpretation of the resistance measurements performed.

The SEM images (Figure 1c,d) show the morphology of the FeSe grains in detail, indicating the shape of the FeSe grains. The optical image gives the impression that the grains would be elongated ones, but from the SEM images, it is clear that the FeSe grains are relatively small platelets with extensions in the nanometer range and form clusters, which are then arranged in a chain-like fashion.

**Figure 2:**
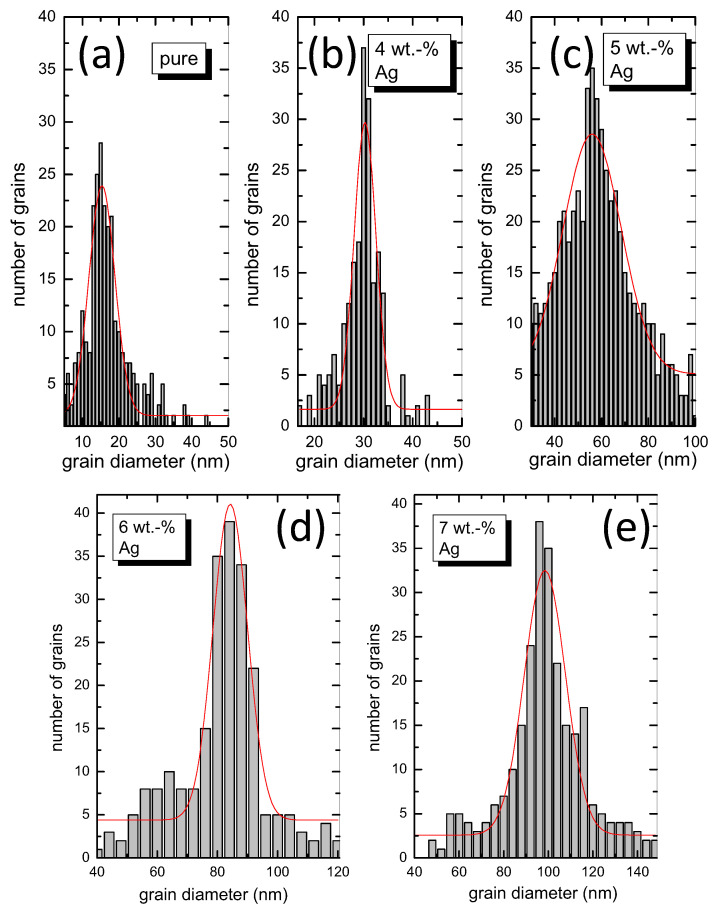
Grain diameter statistics for all five polycrystalline FeSe samples with Ag addition studied here, (**a**) pure, (**b**) 4 wt.-% Ag, (**c**) 5 wt.-% Ag, (**d**) 6 wt.-% Ag, and (**e**) 7 wt.-% Ag.

From SEM images, as well as from AFM topography scans presented in [22], the grain size distribution was determined using the Gywddion software for each of the samples investigated here. This analysis is presented in Figure 2a–e. Gauss fits were employed to determine the mean grain size, which is 15 nm for the pure sample, 30 nm for the 4 wt.% Ag-added product, 56 nm for the sample with 5 wt.% Ag addition, 84 nm for the 6 wt.% Ag added product, and 98 nm for the sample with 7 wt.% Ag addition. The mean grain size increases upon the Ag addition. This will later take a great part in the interpretation of the FIC analysis.

**Figure 3:**
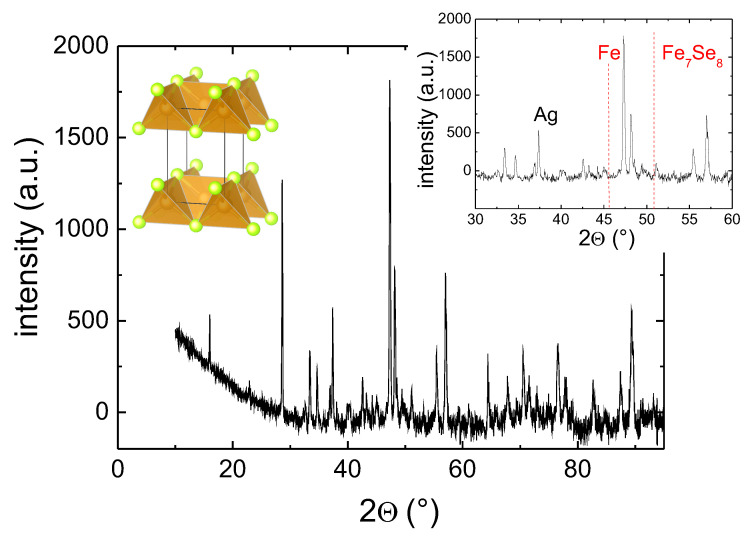
X-ray 2Θ-scan for the sample of 4 wt.% Ag. The left inset gives the crystal structure of β-FeSe, and the right inset shows the peaks at 44.73${}^{\circ }$ and 50${}^{\circ }$, where pure Fe and the Fe${}_{7}$Se${}_{8}$ contribution can be identified following [17].

Figure 3 presents an X-ray 2Θ scan of the sample FeSe + 4 wt.% Ag. All peaks stem from FeSe phases and metallic Ag. The inset to Figure 3 presents a section of the analysis, showing the most informative part between 2Θ= 30 and 60${}^{\circ }$. At 2Θ= 37.4${}^{\circ }$, the Ag-peak is located; just below 45${}^{\circ }$ (44.73${}^{\circ }$), the peak would indicate the presence of metallic, excess Fe, and at 50${}^{\circ }$, the peak would indicate the presence of the antiferromagnetic Fe${}_{7}$Se${}_{8}$. The latter two peaks were discussed by Williams et al. [17], as these two peaks enable judging about the magnetic behavior of the sample (i.e., ferromagnetic (α-Fe) or antiferromagnetic (Fe${}_{7}$Se${}_{8}$). From Figure 3, one can see that there is no contribution of Fe${}_{7}$Se${}_{8}$, but we do have excess α-Fe in the sample, resulting in a ferromagnetic sample at room temperature.

### 4.2. Resistivity Measurements

**Figure 4:**
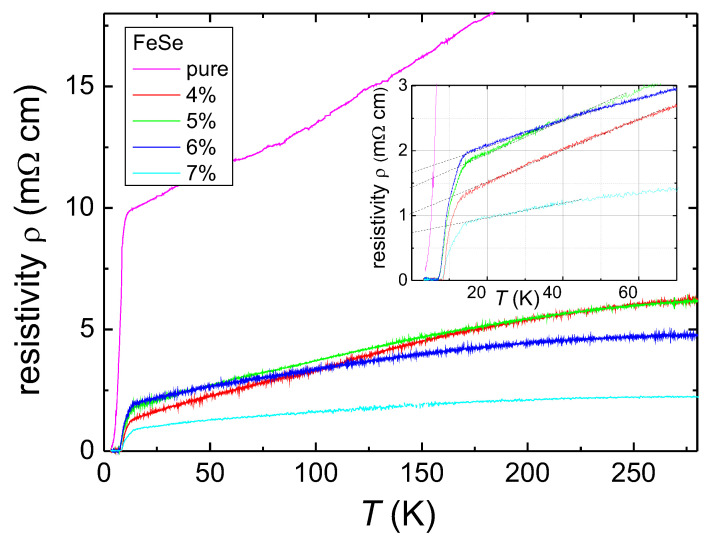
Resistivity measurement on the pure FeSe sample and on the Ag-containing FeSe samples in zero applied magnetic field. The Ag addition reduces the measured resistivity and also affects the superconducting transition. The inset reveals an enlarged view close to the superconducting transition and the determination of the residual resistivity.

**Figure 5:**
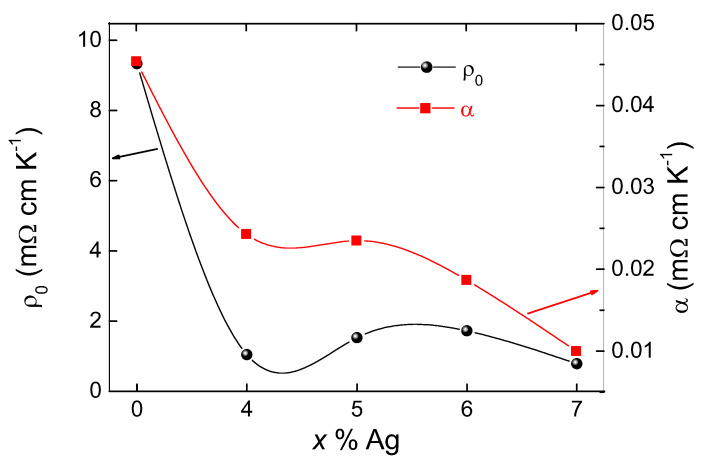
Variation of $\rho_{0}$ and α for the Ag-doped samples with 4, 5, 6, and 7 wt.% Ag additions as determined from the resistivity measurements.

Figure 4 presents the resistivity measurement at zero applied magnetic field for all samples under study. The Ag addition clearly reduces the resistivity and also influences $T_{c}$, as well as the transition width, $\Delta T_{c}$. As already mentioned in [28], the pure FeSe sample shows a small kink in the resistivity data at ∼85 K, whereas this kink completely vanishes in the data of the Ag-containing samples. According to [31], this is an indication that the Ag is not only located at the grain boundaries, but may also intercalate the FeSe unit cell, which would explain the observed changes in $T_{c}$ and $\Delta T_{c}$. As the transport currents through the sample must flow across numerous grain boundaries, around the secondary phase particles, and the still existing pores, the resistance data of the pure sample reflect all these obstacles. The inset to Figure 4 presents the details of the superconducting transition of the Ag-containing samples. The rounding of the superconducting transition is clearly visible, which is in stark contrast to metallic superconductors. The hatched lines indicate the determination of the residual resistivity, $\rho_{0}$. The equation $\rho \left(T\right)=\rho_{0}+\alpha T$ expresses the metallic behavior of the resistivity in the normal state. Here, α describes the intrinsic electronic interaction, and $\rho_{0}$ is determined by the defect density, impurities, and heterogeneities in the sample. Figure 5 presents the data for $\rho_{0}$ and α as determined from the resistivity data. $\rho_{0}$ is clearly highest in the pure sample and reduces considerably for the Ag-doped ones. This decrease of $\rho_{0}$ implies an increase of the charge carrier concentration upon Ag doping, which demonstrates the benefit of Ag doping. Generally, the α parameter is governed by the intrinsic electronic interaction and is found to decrease upon Ag doping in this study.

The superconducting transition temperature, $T_{c,\exp}$, was established using the cross-point of two linear fits to the data as described in [28,32]. The transition width, $\Delta T_{c}$, was decided by considering the criteria of 10–90%. The ratio of residual resistance RRR =R(T= 300 K)/*R*(T= 14 K) was also determined. RRR increases for the 4 wt.% Ag-added product and then reduces with the further increase of the silver content. This tendency is like that observed in [21], except the product added with silver content of 7 wt.%, which shows the optimal values among all prepared samples. The connectivity among grains is also another crucial parameter. The geometrical connectivity could be assessed by employing a procedure that describes various MgB${}_{2}$ products [33]. The connectivity, κ, is determined through:

(3) $$\kappa =\Delta \rho_{g}/\Delta \rho \;\;\;,$$

while Δρ=ρ(300 K)−ρ(14 K) and $\Delta \rho_{g}$ could be presumed as 0.471 mΩ.cm for randomly oriented 3D systems [33]. The various obtained parameters are listed in Table 1.

From the data presented in Table 1, we can conclude that the Ag addition acts in a two-fold manner: (i) There is an increase of $T_{c}$ upon the incorporation of silver in comparison to the pristine sample, and the superconducting transition, $\Delta T_{c}$, becomes sharper. On the other hand, (ii) the normal state resistance, the RRR, and the grain connectivity improve upon Ag addition.

For the FIC analysis, the determination of $T_{c1}$ is required, which is taken as the maximum of the derivative dρ/dT. In the following, we will refer to this temperature as $T_{c}$. The plots of dρ/dT as a function of the applied field were already presented in [28], so we give only the peak positions obtained in Table 1.

### 4.3. FIC Analysis

**Figure 6:**
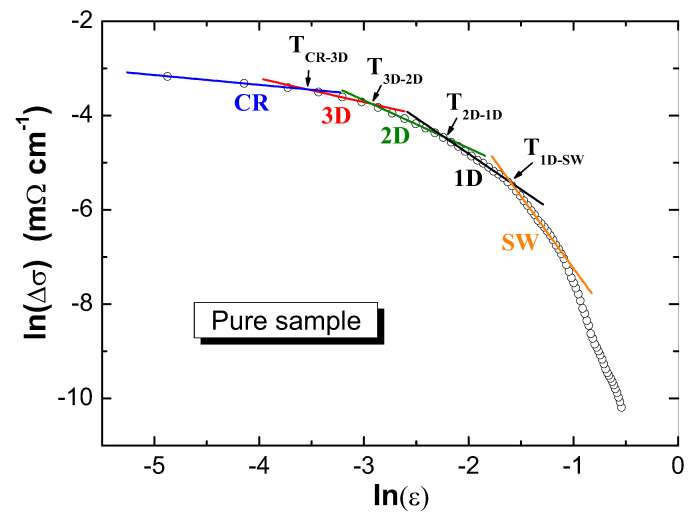
Analysis of the fluctuation-induced conductivity for the pure FeSe sample, using a plot of ln(Δσ) vs. lnϵ. Note the five fluctuation regimes shortwave (SW) (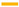), 1D (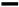), 2D (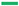), 3D (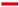), and critical region (CR) (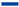) being present.

**Figure 7:**
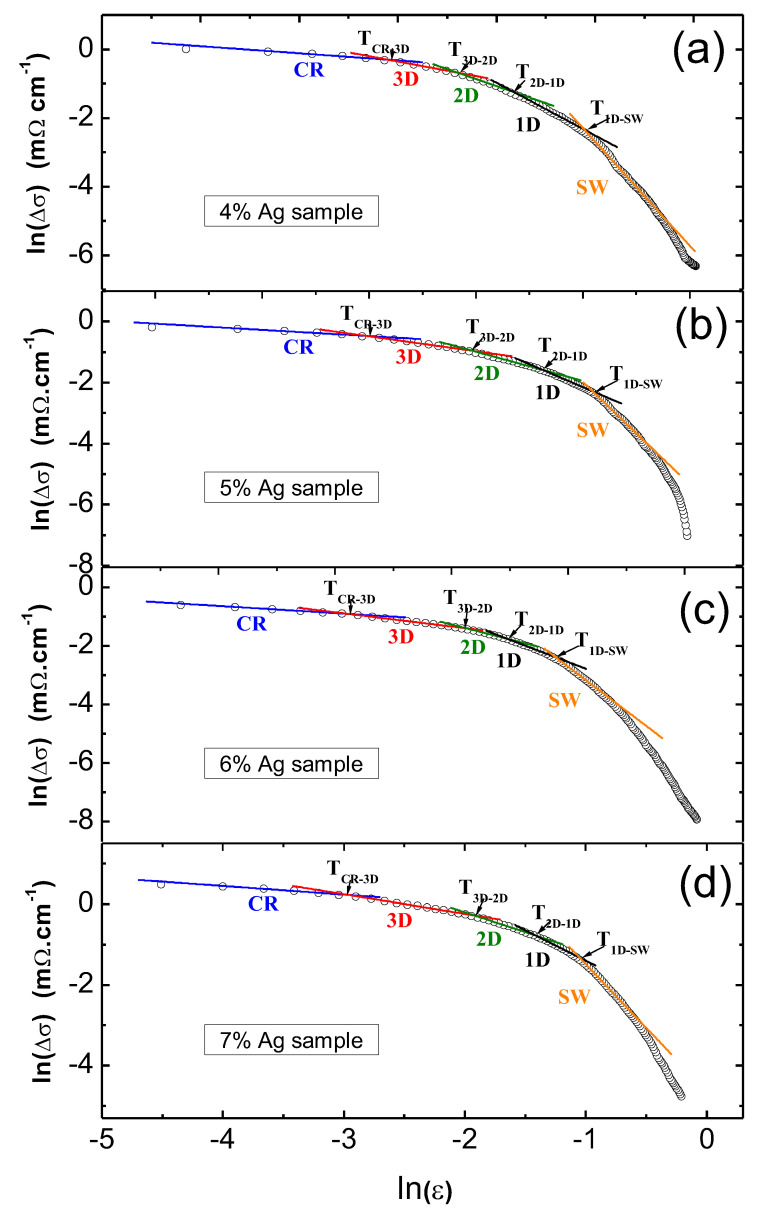
Analysis of the fluctuation-induced conductivity for the Ag-doped FeSe samples with (**a**) 4, (**b**) 5, (**c**) 6, and (**d**) 7 wt.% Ag addition. Note the five fluctuation regimes SW (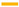), 1D (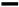), 2D (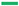), 3D (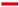), and CR (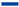) being present.

**Figure 8:**
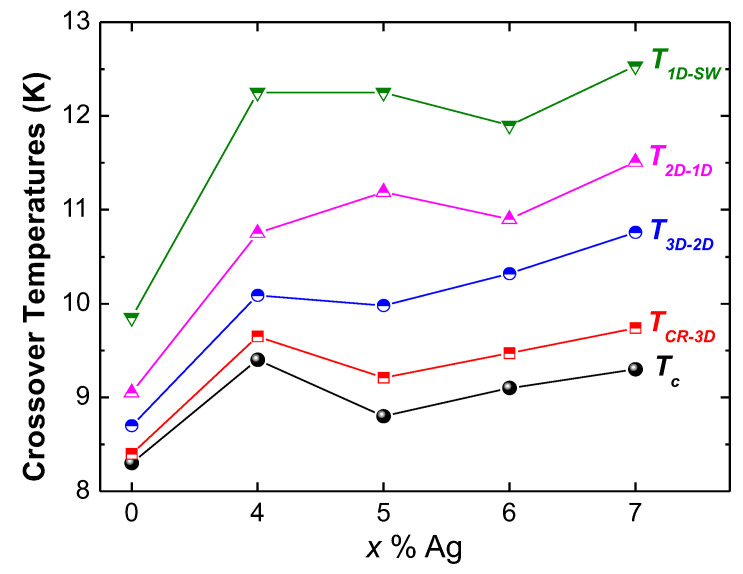
Plot of the various crossover temperatures determined from the FIC analysis as a function of the Ag content, together with the respective $T_{c}$.

The analysis of the FIC enables a deeper insight into what happens around the superconducting transition. Figure 6 presents the results of the FIC analysis on the pure FeSe sample. The plot is divided into five fluctuation regimes, labeled as the CR (=critical region), 3D, 2D, 1D, and SWF (=short wave) fluctuation regimes. Figure 7a–d presents the FIC analysis for the FeSe samples with various additions of silver. The overall behavior of all samples is very similar; also here, five fluctuation regimes are obtained. However, the crossover temperatures of the different regimes and the corresponding widths depend strongly on the Ag content, which is further illustrated in Figure 8. Coming from the pure FeSe sample, the Ag addition leads to a steep increase of $T_{c}$ and the related crossover temperatures, which remain stable or decrease again upon further increasing the Ag content. Here, it should be noted that the entire fluctuation regime in the pure sample extends up to 9.87 K, while the 7 wt.% Ag-added product exhibits an extension of the fluctuation regime up to 12.53 K.

The SWF regime arising at $T\gg T_{c}$ has a dominating role and indicates that the GL theory has ended. This regime develops when ξ and the characteristic wavelength of order parameter are of the same order [23,24]. As a consequence, Δσ reduces quickly in this temperature interval. Upon further reduction of the temperature, a transition from shortwave fluctuations to the mean-field regime (MFR) occurs at $T_{1D-\mathrm{SWF}}$. The MFR is comprised of three distinctive linear parts, where the first part between $T_{1D-\mathrm{SWF}}$ and $T_{2D-1D}$ indicates the presence of 1D fluctuations and the 1D conduction channels. This regime is followed by a 2D fluctuation regime owing to the layered character of the material. A further reduction of the temperature leads to the appearance of another regime (below the temperature $T_{3D-2D}$), which is the 3D regime. Finally, a transition from the 3D fluctuation regime to the critical fluctuation regime is observed at the Ginzburg temperature $T_{G}$. In the CR regime below $T_{G}$, the amplitude of the order parameter and its fluctuations are of a similar size; thus, the GL approach becomes inappropriate.

In the present samples, all five fluctuation regimes can be detected. The determined exponents λ and the widths of various fluctuation regions in the MFR region are listed in Table 2, and the corresponding crossover temperatures are given in Figure 8.

From the models of FIC, it is now possible to obtain several fundamental parameters of superconductivity. The Ginzburg number $N_{G}$ is expressed as follows [26,27]:

(4) $$N_{G}=\left|\frac{T_{G}}{T_{c}}-1\right|$$

The temperature $T_{\mathrm{LD}}=T_{3D-2D}$ is related to the Josephson coupling constant, $E_{J}={\left(2\xi_{c}\left(0\right)/d\right)}^{2}$, by the following expression [34]:

(5) $$T_{\mathrm{LD}}=T_{c}\exp\left[{\left(2\xi_{c}\left(0\right)/d\right)}^{2}\right]\;\;\;,$$

The superconducting parameters, $B_{c1}\left(0\right)$, $B_{c2}\left(0\right)$, and $J_{c}\left(0\right)$, are deduced by the following expressions [26,27]:

(6) $$B_{c}\left(0\right)=\frac{\Phi_{0}}{\pi \sqrt{8}\xi_{c}\left(0\right)\lambda \left(0\right)}\;\;,$$

(7) $$B_{c1}\left(0\right)=\frac{\ln\kappa }{\sqrt{2}\kappa }B_{c}\left(0\right)\;\;,$$

(8) $$B_{c2}\left(0\right)=\sqrt{2}\kappa B_{c}\left(0\right)\;\;,$$

(9) $$J_{c}\left(0\right)=\frac{4\kappa }{3\sqrt{3}\ln\kappa \lambda \left(0\right)}B_{c1}\left(0\right)\;\;,$$

where $B_{c}\left(0\right)$ is the thermodynamic critical field, $\Phi_{0}=\frac{h}{2e}=$ 2.07 × 10${}^{-15}$ T/m${}^{2}$ denotes the flux quantum, λ(0) is the penetration depth at 0 K predicted by London (considered as 325 nm, [35]), and κ=λ/ξ is the GL parameter. All the superconducting parameters estimated from the FIC analysis are reported in Table 3.

The mean grain size of the FeSe samples was found to increase upon Ag addition as described in Section 4.1. According to previous studies [36], this indicates a possible increase or enlargement of the width of the 3D fluctuation region and a reduction in the width of the 2D fluctuation region. In the present investigation, the width of the 3D fluctuation regime (see Table 2) matches very well with this tendency, whereas the width of the 2D fluctuation regime fluctuates.

Now, we can discuss the superconducting parameters obtained from the FIC analysis.

The Josephson coupling, $E_{J}$, slightly decreases upon Ag addition from about 0.64 to 0.54, which is distinctly different from materials like Bi-2212 and Bi-2223, where $E_{J}$ is found around 0.025 [37,38]. In Ag-added YBa${}_{2}$Cu${}_{3}$O${}_{y}$ (YBCO), $E_{J}$ was found to be in the range of 0.30–0.35 [39], which is two times lower than the values obtained here. The observed reduction of the $E_{J}$ value upon Ag addition suggests the intensification of the density of charge carriers within the conducting planes.

**Figure 9:**
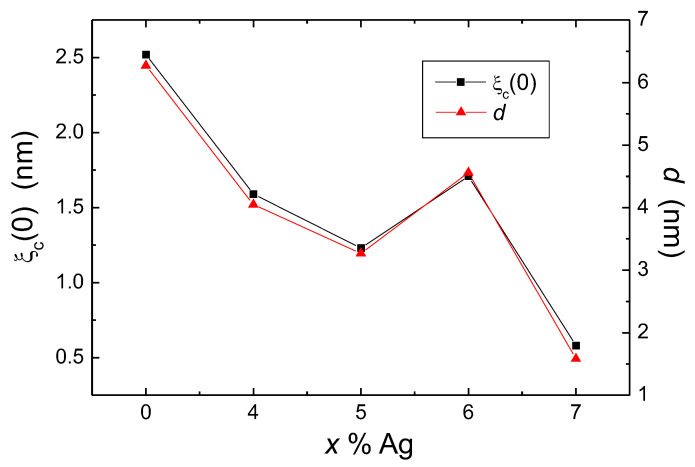
Superconducting coherence length, $\xi_{c}\left(0\right)$, and thickness of the 2D fluctuation layer, *d*, as a function of the Ag addition.

Figure 9 shows the variations of $\xi_{c}\left(0\right)$ and *d* with Ag content in the FeSe samples as determined from the FIC analysis. The coherence length for the pure FeSe sample determined here compares well to the data published in [40,41] for FeSe single crystals ($\xi_{c}\left(0\right)=$ 2.9 nm). Therefore, we see that the Ag addition to FeSe reduces $\xi_{c}\left(0\right)$ and consequently enhances also $B_{c}\left(0\right)$ and $B_{c2}\left(0\right)$, which is an additional benefit. With the exception of the sample with 6 wt.% Ag addition, $\xi_{c}\left(0\right)$ decreases with increasing Ag content. The lowest values of $\xi_{c}\left(0\right)$ together with the variation of $E_{J}$ in the Ag-added FeSe samples reflect a growth in the charge carrier density within the conducting planes in comparison to the pristine product. The *d* parameter ranges between 6 and 2 nm and also decreases with increasing Ag content. The *d*-values obtained here are much smaller than for other materials studied before (e.g., YBCO, Bi-2212, Bi-2223), which indicates a strong localization of the fluctuations into a narrow plane, owing to the crystallographic structure of FeSe.

In [40,41], the anisotropy parameter, γ, was determined to be 2.4 for FeSe. Taking this value, we find $\xi_{ab}\left(0\right)=$ 6.04 nm for the pure FeSe samples, which compares well to the 5.2 nm found in [40]. The values for the thermodynamic critical field, $B_{c}\left(0\right)$, obtained here are reasonable for polycrystalline HTSc samples. Here, it is obvious that the pore-filling effect of the Ag addition considerably improves $B_{c}\left(0\right)$, which can reach a value of 1.22 T for the 7% Ag-added product. The data for the lower critical field, $B_{c1}\left(0\right)$, are found in the millitesla regime. The $B_{c2}\left(0\right)$ data were obtained from Equations (6)–(9), which are based on the Werthamer–Helfand–Hohenberg (WHH) theory [42]. In [40], the fits to the data obtained with a resonance frequency technique led also to values above 40 T for $B_{c2}\left(0\right)$. Regarding the spin-paramagnetic effect, much smaller values for $B_{c2}\left(0\right)$ of about 12 T (H‖c) and 29 T (H⊥c) were obtained, which fit much better to other published measurements and to values obtained on other IBS materials. Therefore, this spin-paramagnetic correction is also required for our analysis. This is a clear hint that the magnetic configuration (i.e., the Fe itself and the embedded magnetic particles of α-Fe and Fe${}_{7}$Se${}_{8}$) of the FeSe samples plays a significant role in the superconducting characteristics of samples, especially in applied magnetic fields. However, the comparison of the present data shows that all the Ag-containing samples also have much better $B_{c2}\left(0\right)$ values as compared to the pure FeSe sample.

These observations are again proof that Ag may intercalate the FeSe unit cell and, thus, alter the superconducting parameters. Such intercalation of FeSe was found originally using potassium [43] and was reviewed recently in [44]. These materials may reach a $T_{c}$ of ∼ 30 K. This may also be possible with the addition of Ag. Our data show clearly that all Ag-containing FeSe samples are better and stronger superconducting materials compared to the pure FeSe samples fabricated by solid-state sintering. We can directly compare this situation with the Ag (or Ag${}_{2}$O) addition to the YBa${}_{2}$Cu${}_{3}$O${}_{y}$ compound, which was extensively studied in the literature (see, e.g., [45,46]). The fluctuation-induced conductivity of such samples was also analyzed in [26,39]. In this case as well, the upper critical field increased by a factor >2 upon Ag addition, and the critical current improved by a factor >3. The superconducting transition temperature, however, remained unchanged or even decreased upon Ag addition [39]. These observations demonstrate again that the Ag addition to FeSe does not just fill the pores and reduce the electric resistance, but also alters the electronic configuration.

From the present analysis, it could be concluded that the FeSe product added with a silver amount of 7 wt.% turns out to exhibit the best superconducting properties of the entire series of samples regarding the electric/resistive properties. In contrast to this, the sample with the 4 wt.% Ag addition was found in [28] to exhibit the best flux pinning properties. Thus, the optimum amount of Ag to be added to FeSe strongly depends on the demands of the given application. Overall, the superconducting properties of the FeSe system were enhanced upon the inclusion of silver.

## 5. Conclusions

The microstructure and the fluctuation-induced conductivity were investigated for a series of polycrystalline FeSe superconductors with silver incorporation. The mean grain size of the FeSe samples was established to increase upon Ag addition. The Ag fills the pores between the grains, improves the connectivity and resistivity, as well as affects the superconducting properties of the FeSe phase as indicated by the changes of $T_{c}$, $\Delta T_{c}$, and the crossover temperatures of the various fluctuation regimes. Using the fluctuation-induced conductivity analysis, five fluctuation regimes with their exponents and crossover temperatures were recognized. From this information, we determined several superconducting parameters of the pure FeSe sample and a series of FeSe samples with the addition of metallic silver. Together with the microstructure analysis, important information on the Josephson coupling of the FeSe grains, the layer thickness of the 2D fluctuations, and the width of 2D and 3D fluctuation regimes, which are directly affected by the mean grain size, is found. The sample with the 7 wt.% Ag addition turns out to be the one with the best superconducting parameters. Thus, we can state that the Ag addition to FeSe has a great impact on improving the superconducting parameters as determined by the FIC analysis.

## Figures and Tables

**Table 1:** Superconducting and electric parameters of the studied FeSe products. RRR, ratio of residual resistance.

Product	Ag-Content	$T_{c,\exp}$	$\Delta T_{c}$	$T_{c1}$	*R* (300 K)	RRR	Grain Connectivity
		(K)	(K)	(K)	(Ω)		(%)
1	pure	8.35	2.35	8.33	0.03224	2.16	3.9
2	4 wt.%	9.42	0.98	9.11	0.00930	4.49	6.52
3	5 wt.%	9.28	1.18	8.64	0.00926	3.33	7.3
4	6 wt.%	9.36	1.12	8.47	0.00718	2.41	11.3
5	7 wt.%	9.18	0.94	8.57	0.00335	2.44	23.8

**Table 2:** The conductivity exponents λ and the width of the mean-field regime (MFR) fluctuation regimes (3D, 2D, and 1D) as function of the Ag addition.

Sample	$\lambda_{\mathrm{CR}}$	$\lambda_{3D}$	$\lambda_{2D}$	$\lambda_{1D}$	$\lambda_{\mathrm{SW}}$	3D Width	2D Width	1D Width
						(K)	(K)	(K)
pure	0.21	0.50	1.01	1.51	3.04	0.3	0.35	0.8
4 wt.%	0.21	0.47	1.03	1.53	3.16	0.44	0.66	1.5
5 wt.%	0.20	0.49	1.04	1.50	3.36	0.77	1.21	1.06
6 wt.%	0.24	0.48	1.08	1.52	3.03	0.85	0.58	1
7 wt.%	0.21	0.48	0.98	1.50	3.15	1.02	0.75	1.02

**Table 3:** Superconducting parameters of FeSe samples with silver addition determined from the fluctuation-induced conductivity (FIC) analysis.

Sample	$N_{G}$	*d*	$E_{J}$	κ	$\xi_{c}\left(0\right)$	$B_{c}\left(0\right)$	$B_{c1}\left(0\right)$	$B_{c2}\left(0\right)$	$J_{c}\left(0\right)$
	× 10${}^{-2}$	(nm)			(nm)	(T)	(× 10${}^{-3}$ T)	(T)	(× 10${}^{5}$ A/m${}^{2}$)
pure	1.20	6.27	0.64	128.83	2.52	0.28	7.57	51.73	4.76
4 wt.%	2.65	4.05	0.62	203.38	1.59	0.45	8.28	128.94	7.51
5 wt.%	5.00	3.27	0.56	263.97	1.23	0.58	8.69	217.21	9.74
6 wt.%	4.06	4.56	0.57	189.25	1.71	0.41	8.17	111.65	6.98
7 wt.%	4.73	1.58	0.54	553.29	0.58	1.22	9.87	954.33	20.43
